# The COPE LBP trial: Cognitive Patient Education for Low Back Pain - a cluster randomized controlled trial in primary care

**DOI:** 10.1186/1471-2474-11-33

**Published:** 2010-02-16

**Authors:** Erik L Werner, Kjersti Storheim, Ida Løchting, Margreth Grotle

**Affiliations:** 1 Research Unit for General Practice, Uni Health, Bergen, Norway; 2 FORMI - Communication unit for musculoskeletal disorders, Oslo University Hospital, Oslo, Norway

## Abstract

**Background:**

Non-specific low back pain (LBP) is usually self-limiting within 4-6 weeks. Longstanding pain and disability are not predictable from clinical signs or pathoanatomical findings. Pain cognition and physical performance have been shown to improve patients with chronic LBP following neurophysiological education. The primary aim of this study is to evaluate whether a specific cognitive based education programme for patients with LBP in primary care is more effective than normal care in terms of increased function. The secondary aims of the study are to evaluate whether this intervention also results in earlier return to work, decreased pain, increased patient satisfaction, increased quality-of-life, and cost utility.

**Methods/Design:**

Cluster randomised controlled trial with 20 general practitioners and 20 physiotherapists in primary care as the unit of randomisation. Each practitioner will recruit up to 10 patients, aged 20 to 55 years, with non-specific sub-acute/chronic LBP of more than four weeks but less than 1 year's duration. Practitioners in the intervention arm will provide cognitive patient education intervention in up to four weekly sessions, each lasting 30 minutes. Practitioners in the control arm will provide normal treatment, but have to make four appointments for the patients. Patients, outcome assessors, and study statistician will be blinded to group allocation.

**Discussion:**

We present the rationale and design of an ongoing RCT study that potentially offers an easily implemented treatment strategy for LBP patients in primary care. The results will be available in 2012.

**Trial registration:**

ISRCTN04323845

## Background

LBP is a major health problem in the society. The lifetime prevalence of LBP is estimated to 70% [1], the one-year prevalence in Norway 53% [2] and two weeks prevalence 42% [3]. The direct and indirect annual cost in Norway due to LBP is estimated to 15 Billion NOK, and 16% of long-term sickness absence and 16% of new disability claims have LBP as a primary diagnosis [4]. LBP patients are also heavy health care consumers constituting 5-10% of all family doctors' consultations [5,6], 27% of the physiotherapists' treatment and 82% of the chiropractors' treatment [5,6].

As most acute LBP is self-limiting within few weeks [1], only 10% of the patients are referred from their family doctor to specialized health care [4]. However, about 15% of acute LBP proceed to a chronic stage (duration > 3 months), and 73% will have one or several recurrences within a year [7]. An interesting feature of LBP disability is that 75% or more of the costs of LBP can be attributed to 5% of the patients. The decisive factors for future disability do not appear to be any characteristics of the LBP; but are primarily psychosocial factors [8].

Frustration arises because of the lack of consistency among the professionals regarding treatment and understanding of the problem [9,10]. Unfortunately, the Norwegian guidelines do not seem to have had great impact on the health care providers' practices [11,12]. Specific strategies for implementing guidelines in primary care have not shown changes in patients' sickness behaviour either [13,14].

The view on non-specific LBP has changed during the past two decades from a purely "injury model" to a "biopsychosocial" understanding of the condition [15,16]. In this model, the back pain arises from nociception of pain in the back caused by reasons or tissue injury that cannot be identified. The pain may result in varying degrees of dysfunction, not necessarily only related to the magnitude of the injury, but also to how the pain is perceived. The second element of this model is how the patients think and feel about the dysfunction, thus determining how it affects them. This involves beliefs and coping strategies. The degree of anticipation, anxiety, attention, and previous experiences reflect our perception of the pain, leading in turn to beliefs that determine how we manage to cope with the actual pain.

Based on this understanding, both international and Norwegian guidelines recommend cognitive intervention as an essential element in the treatment of long-lasting LBP [1]. A recent systematic review on patient education programmes for chronic LBP (such as back schools, brief education, and fear-avoidance training) also recommended brief education programmes in clinical settings [17].

We thus have sufficient knowledge about management of LBP, which should theoretically reduce the consequences of back pain to a minimum, but so far different strategies for implementation of this knowledge have failed to produce the desired results [18]. There is limited knowledge on effective ways to change clinical practice. In a recent systematic review of 235 studies on guideline dissemination and implementation strategies, the authors found more evidence for clinician-oriented interventions (e.g. use of education, reminders, feedback) than for intervention aimed at the organisation or the patient [19]. Further, they found very little information on patients' outcomes or financial assessment of the implementation strategies.

A mass media campaign has previously been conducted in Norway, which aimed at changing public beliefs and following health behaviour in back pain patients [20]. Unlike an Australian campaign [21], but in line with campaigns in Scotland and Canada [22,23], the Norwegian campaign did not succeed in changing sickness behaviour in terms of sick leave, surgery or the use of imaging scans in patients with LBP.

Other previous randomised controlled trials designed to implement LBP guidelines (and change the behaviour of clinicians) have used different intervention including educational outreach, multi-faceted intervention with workshops and printed educational material, dissemination, and audit and feedback [24,25]. The effectiveness of these trials varied with regard to the success of changing the behaviour of the clinicians [26]. However, these studies did not include measurement of patient outcomes or cost-effectiveness analysis.

One of the key messages in clinical guidelines for LBP is the advice to remain active in order to prevent a chronification of the LBP problem. However, there is little theoretical basis for this advice. In this trial we want to implement a cognitive based education programme, which is theoretically based on current understanding and knowledge of pain mechanisms. The primary aim of this study is to evaluate whether a specific cognitive based education programme for patients with low back pain in primary care is more effective than normal care in terms of increased function. The secondary aims are to evaluate whether this intervention also results in earlier return to work, decreased pain, increased patient satisfaction, increased quality-of-life, and cost utility.

Our main hypothesis is that this cognitive intervention will result in higher physical function compared with normal care. We also hypothesise that cognitive intervention results in decreased pain, more satisfied patients, better quality-of life, and is more cost-effective than normal care.

## Methods/Design

### Design

This study is a stratified cluster randomised controlled trial taking place in primary care with family doctors (GP) and physiotherapists (PT) in Oslo and Akershus County in Norway. The design contains two arms in which 20 GPs and 20 PTs will be stratified into, and then randomised to either the intervention or the control provider group. Each cluster will contain the GPs/PTs and their included patients.

### Recruitment of practitioners

An invitation to an information meeting regarding the project was sent out to all GPs and PTs working in the primary health care in Oslo and Akershus County in Norway in March 2008. After an information meeting, the practitioners were invited to participate in the study. A total of 20 GPs and 20 PTs were included and randomised.

Strategies to promote participation of practitioners into the trial included offering professional development points and financial compensation for the extra time spent on recruiting patients to the project, as well as treatment of included patients.

### Recruitment of patients and eligibility criteria

Each participating GP and PT in both intervention and control groups will consecutively ask eligible patients to participate in the study. The study is thus taking place in a normal clinical setting. The inclusion criteria are:

• Men and women aged 20 - 55

• Unspecific LBP

• Duration from 4 weeks up to 1 year since onset of actual pain episode

• Signed informed consent

• A score ≥ 4 on the Roland Morris Disability Questionnaire

The following criteria excluded patients from participation:

• Provider's uncertainty of diagnosis; possible nerve root pain or severe pathology

• Signs of any 'red flags'

• Particular interest in or demand for a specific treatment

Consequently, all patients visiting his or her provider because of back pain will be assessed according to these inclusion and exclusion criteria. If the patient is found eligible, he or she will be asked to participate. Those who agree to participate will sign a consent form and receive the questionnaires described later. The patients may either answer the questionnaires immediately in the provider's waiting room, or at home. In both cases the forms will be sent in a stamped and addressed envelope before the first session of the trial. The provider will inform the study management by an enrolment fax and arrange for the first session within a week of inclusion. Those who do not want to participate in the study or fail to satisfy the criteria will be registered by initials, year of birth, score on the Roland Morris Disability Questionnaire and the reason for not participating. All the procedures are identical for both intervention and control groups.

### Randomisation and allocation procedure

GPs and PTs were randomly allocated to either the intervention or control group. A block randomisation procedure with stratification for type of health care provider (GP and PT) was carried out to ensure a similar number of participants in the four blocks (GP and PT intervention, GP and PT control). The randomisation procedure was carried out by computer-generated numbers that were placed in closed non-transparent envelopes. An independent person administrated this procedure. Patients and outcome assessors will be blinded regarding group allocation.

### Intervention group

The professionals have been trained in the specific model of LBP treatment based on the biopsychosocial understanding of LBP and the "intensive neurophysiology education". Following this training they will provide their LBP patients in their normal clinical settings with a four-session treatment programme consisting of this education programme in addition to their normal treatment. At each treatment session the caregiver will stick to the procedures described in detail in the trial manual when communicating with the patient.

The "intensive neurophysiology education" programme has been developed by a group of British and Australian researchers as an education programme for patients with LBP. The cognitive elements of this programme consist basically of an understanding of pain that differs somewhat from the traditional "injury model". This theory is primarily based on the neurophysiology of pain, reflected by sensitisation and neuronal response to inactivity and movement control [27]. Based on this, the education programme has three basic elements [28]:

• Reduction of what the patients perceive as threatening inputs to the brain

• Targeting the patients' own understanding of the pain

• Exposure to the threatening inputs

Previous studies have documented an additional effect of "intensive neurophysiology education" when combined with physiotherapy [27].

The intervention will be given as four 30-minute sessions once a week for four consecutive weeks. This treatment is specified in a manual for this trial and all intervention doctors and physiotherapists have attended the 18 lessons course. They are also provided with a written summary of the content of each session.

Each of the sessions has a specific content of education and discussion in the one-to-one setting between the provider and the patient. Initially the discussion concerns the thoughts and fears of the patients and their LBP. Eventually, the education programme will deal more and more with exposure to movements and daily activities that the patients more or less deliberately avoid because of fear of provoking pain. As homework between the sessions, the patients will be asked to identify barriers to normal functioning, reasons for fear avoidance behaviour and other reflections related to the education. They will also be asked to make specific registrations regarding function, pain and work absence.

### Control group

The providers of the control group are asked to register and include all patients meeting the inclusion criteria in a similar manner. These patients will also answer on all questionnaires, and will meet their provider weekly for up to four weeks. These sessions have no defined content, but the providers will spend somewhat more time on these patients than the regular schedule, asking for more details on what prevents them from resuming normal activity. The control group providers have not participated in the training course of the intervention and therefore their care will consist of whatever is the normal procedure for the individual doctor or physiotherapist, only that the time spent for each patient will be somewhat longer than normal (particularly for the doctors). This is done to minimise the effect of the increased attention in itself so that any observed differences between the groups are more likely to be due to the "intensive neurophysiology education".

### Data collection

Patients will initially be asked by the provider to answer the questionnaires in the waiting room immediately after signed consent. At baseline the patients will fill in a registration form and a baseline questionnaire regarding sociodemographics (age, gender, marital status, education, and work status), other diseases, use of medicines, a standardised screening questionnaire on psychosocial risk factors - the Örebro Musculoskeletal Pain Questionnaire [29,30] and the baseline measures of the outcomes. In addition, three standardised questionnaires assessing beliefs about pain, the Pain Catastrophizing Scale [31], the brief version of the Illness Perception Questionnaire [32,33], and beliefs about LBP [20] will be included. The two first questionnaires will be translated into Norwegian and tested for psychometric properties before start of the main study.

During the first four weeks, all the patients will fill in a weekly report regarding work status and pain rating. The patients will be contacted again after 4-5 weeks, and after 3, 6, and 12 months after inclusion. At 4-5 weeks, 3 and 6 months, the patients will be contacted by telephone for the registration of work status and pain rating, and at 4-5 weeks and 12 months follow-up the patients will also fill in a postal comprehensive questionnaire including outcome measures and questions about possible co-intervention.

### Outcome measures

Outcome measures will be recorded by an assessor blinded to group allocation. The primary outcome is *function (disability) *assessed by the Roland Morris Disability Questionnaire (RMQ) [34-36], a self-administered questionnaire with score 0-24, with 0 corresponding to no disability. The RMQ is a frequently used outcome measure in trials related to LBP.

The secondary outcomes are as follows:

• *Pain; *measured on a numerical rating scale from 0 to 10

• *Return to work; *reported as number of days from first visit to the health care provider until complete or partial return to work - and as a total number of days absent from work the following year

• *Overall satisfaction*; measured on a numerical rating scale from 0 to 10

• *Health related quality of life*; assessed by the EuroQoL-5D [37] and Patient-Generated Index (PGI) [38]

• *A cost-effectiveness analysis *will be carried out by using both the return-to work outcome and the EuroQoL 5D, and by estimating the total cost of all the four groups of doctors and physiotherapists in this trial.

### Compliance

In order to assess compliance with the treatment intervention, three initiatives are taken: Firstly, the intervention clinicians filled in a knowledge test before and after the 2-day seminar with Lorimer Moseley. This test has been developed by David Butler and Lorimer Moseley, and is frequently used when providing the "explain pain" courses. The test will be used again in one of the follow-up meetings throughout the project period.

Secondly, in order to evaluate the clinician's perception of the intervention he/she has provided, the clinicians reply to some questions regarding the content of the provided intervention for each of the patients included.

Thirdly, in order to evaluate the patient's perception of the intervention he/she has received, a telephone interview after the last treatment session is carried out. All included patients (regardless of group allocation) are asked identical questions.

### Data analysis

A daily checking for accuracy and completion of data forms and double data entry will be used to ensure quality of the data. Data will be analysed by a statistician who is blinded to group status. The primary analyses will be by intention-to-treat and we will restrict the number of analyses in order to reduce the possibility of Type I errors. For primary outcomes, a p value of < 0.05 will be considered statistically significant. For the secondary outcomes a p value of < 0.01 will be considered statistically significant.

Differences between the groups will be presented as a mean with 95% CI or in categories with odds ratio for categorical data. For the primary outcome of days to return to work a survival curves analysis with a log-rank statistic will be used to assess differences between the groups, and Cox's regression to assess the effects of treatment (group) status on hazard rates for time to return to work. A mixed model with group as a fixed factor will be used for the other outcome measures. If there is a significant difference between treatment groups, post-hoc analyses will be conducted. We will also test for potential confounding factors in these models. Analyses of prognostic factors will be carried out by multivariate regression models.

### Sample size

The primary outcome of the study is patient-reported function as measured on a continuous measure (Roland- Morris Questionnaire). Based on previous Norwegian studies using the RMQ for patients with LBP, a clinically relevant difference between two groups can be estimated to be approximately 2 points with a SD of 3. This is also similar to a previous evaluation study of the "intensive neurophysiology education" carried out by Moseley et al [27]. We put the significance level (alpha) to 5% with the probability of at least 80% to discover a difference between the two intervention groups. If the difference between mean changes in the two groups is at least 10% (approximately 2-3 points) with SD of 3, we will need approximately 50 patients in each group. Adjusting for the cluster design of 20 different clusters and loss to follow-up we end up with 150 patients in each group, total n = 300 patients, that is 75 patients in each block. It seems from this sufficient if each provider recruits 7 or 8 patients each in order to obtain 300 in total.

## Discussion

We present the rationale and design for an RCT examining the effects of a specific cognitive based education model for management of sub-acute LBP in normal primary care settings. The primary outcome will be disability as measured by Roland-Morris Disability Questionnaire. The secondary outcomes include pain, return to work, patient satisfaction, quality-of-life, and cost utility. Data collection will take place at inclusion, at the end of the four-week treatment programme and at 12 months. The study period will go through 2009 and 2010 and the results will be available in 2012.

If the study succeeds in demonstrating a significant positive effect on any of the outcome measures, the potential gain is huge, not only for the LBP patients, but also for the societal costs and for the health care, because of the large number of patients and their disability.

## Ethical considerations

Ethical approval has been gained from the Norwegian Regional Committee for Medical Research Ethics, the Data Inspectorate, and the Norwegian Board of Health (January 2009). The investigators will ensure that the trial will be conducted in compliance with this protocol.

## Competing interests

The authors declare that they have no competing interests.

## Authors' contributions

ELW, KS and MG conceptualised and designed the study, including writing the protocol. Statistician Leif Sandvik, Oslo University Hospital, contributed in the calculation of study sample and will contribute in the statistical analyses. IL has contributed in the methodological study as well as in the pilot study prior to this trial. IL will be the research coordinator who is responsible for the daily checking for accuracy and completion of data forms. An independent person will carry out the telephone interviews and enter the data in the project. All authors contributed in arranging the intervention course for the practitioners included in the intervention group. All authors are also responsible for the written/oral information as well as follow-up meetings for all practitioners participating in the trial. ELW and MG wrote the first draft of this manuscript, whereas KS and IL commented on the draft. All authors have read and approved the final manuscript.

## Pre-publication history

The pre-publication history for this paper can be accessed here:

http://www.biomedcentral.com/1471-2474/11/33/prepub
